# Structural Barriers and the Future of AI in Global Health: Lessons From Eradicable Diseases

**DOI:** 10.7759/cureus.92061

**Published:** 2025-09-11

**Authors:** Kirubel T Hailu, Ryan R Haddad

**Affiliations:** 1 Public Health, University College Cork, Cork, IRL; 2 Clinical Research, California Institute of Behavioral Neurosciences & Psychology, Fairfield, USA

**Keywords:** artificial intelligence in medicine, diseases of poverty, eradication therapy, global health equity, health-care systems, health policy and economics

## Abstract

Despite unprecedented scientific and technological capacity, a substantial burden of eradicable and highly reducible diseases of poverty persists in low- and middle-income countries. These conditions continue to cause preventable mortality and morbidity. While artificial intelligence (AI) is widely promoted as a transformative solution to global health challenges, historical evidence demonstrates that technological innovation alone cannot overcome entrenched political, governance, and equity barriers.

This narrative review synthesizes evidence from peer-reviewed literature, global health agency reports, and policy analyses to identify conditions that could be eliminated or significantly reduced using existing interventions.

Diseases were classified by eradication feasibility and burden, with data compiled on disability-adjusted life years, eradication or control costs, and documented barriers to implementation. Quantitative findings were integrated with a thematic analysis of structural obstacles.

Two primary categories emerged. The first includes eradicable diseases such as poliomyelitis, measles, rubella, malaria, tuberculosis, lymphatic filariasis, obstetric fistula, and severe acute malnutrition. The second comprises highly reducible or preventable conditions, including cervical cancer, hepatitis B and C, maternal mortality, neonatal deaths, diarrheal and respiratory infections, mother-to-child HIV transmission, rheumatic heart disease, and cataract blindness. Proven, cost-effective interventions exist for all of these conditions. However, their implementation is persistently hindered by weak health systems, inequitable funding, fragmented governance, and sociocultural barriers.

The estimated costs of eradication are modest compared with global expenditures in other sectors. This underscores that political will, rather than technological innovation, is the decisive determinant of progress. The persistent underutilization of current tools is the strongest predictor of how future innovations, including AI, will be deployed. Without structural reforms to strengthen health systems, ensure equitable resource distribution, and embed accountability, innovations, including AI, will not succeed where existing solutions have failed. This review calls for reframing disease eradication as a political and moral imperative, prioritizing governance and system capacity alongside technological innovation to achieve equitable health outcomes.

## Introduction and background

Despite unprecedented advances in medical science and technology, a substantial burden of preventable morbidity and mortality persists in low- and middle-income countries (LMICs). Noncommunicable diseases (NCDs) now account for the majority of premature adult deaths in these settings. Over 85% of premature NCD-related mortality occurs in LMICs [1,2]. Although many conditions classified as diseases of poverty, those eradicable or highly reducible with existing interventions, have well-established prevention and treatment options, their deployment remains inconsistent and incomplete [3]. This disconnect between capacity and outcome points to deep structural barriers, particularly inadequate political will, inequitable funding, and weak governance systems [2,4].

Artificial Intelligence (AI) has recently been heralded as a transformative tool for global health, with applications ranging from outbreak detection and surveillance to diagnostic support and resource optimization [5,6]. However, history offers a cautionary perspective: previous technological breakthroughs, such as antibiotics or genomic diagnostics, have failed to yield equitable impact when implemented in structurally weak health systems [7]. Without sustained political commitment, inclusive governance, and robust delivery systems, technological capacity alone has not guaranteed improved population health outcomes [7]. The persistence of eradicable diseases in the 21st century is both a public health failure and a moral indictment of current global health priorities.

The so-called “10/90 gap,” first highlighted by the Global Forum for Health Research to emphasize that under 10% of health research funding addresses conditions responsible for over 90% of preventable deaths in LMICs, remains deeply relevant [8,9]. Recent data from the WHO Global Observatory on Health R&D report that in 2020, low-income countries received a mere 0.2% of all health research grants (including 0.2% for NCDs and 0.6% for neglected tropical diseases) [10]. Research capacity remains starkly imbalanced: high-income countries now have approximately 56 times more health researchers per capita than their low-income counterparts [10]. The most striking inequity is observed in active cancer clinical trials: high-income countries host about 7.05 trials per 100,000 prevalent cases (and 60.02 per 100,000 deaths), compared to only 0.15 trials per 100,000 cases (0.43 per 100,000 deaths) in low-income countries, over a 100-fold difference in trial activity relative to disease burden [11]. The historical underutilization of proven tools serves as a critical predictor of how future innovations, including AI, are likely to be deployed. If existing interventions cannot be delivered effectively, the arrival of more advanced technologies will not, on their own, resolve the underlying inequities that determine who benefits from innovation [7,12].

The research questions guiding this review are as follows: (1) Which diseases of poverty are technically eradicable or highly reducible with existing interventions? (2) What structural, political, and governance barriers have historically limited the effectiveness of these interventions? (3) How might the current wave of AI-based health innovations overcome or replicate these barriers?

This review examines the intersection of technological capacity and political will in eliminating diseases of poverty. It identifies conditions for which eradication or substantial reduction is technically feasible, synthesizes evidence on the costs and feasibility of implementation, and analyzes governance, equity, and system-level barriers that continue to impede progress. Drawing lessons from historical experience, it assesses the likely trajectory of AI in global health and proposes structural reforms to ensure that technological advances translate into equitable health outcomes.

## Review

Methods

Study Design

This study adopts a narrative review approach to synthesize evidence on diseases of poverty that are eradicable or highly reducible using existing interventions. The analysis integrates quantitative data on disease burden and eradication costs with qualitative insights from policy and governance literature, situating technological capacity within broader structural determinants of health outcomes [13,14].

Data Sources and Search Strategy

Peer-reviewed literature was identified through searches of PubMed, Web of Science, and Scopus using combinations of keywords, including eradication, diseases of poverty, global health equity, artificial intelligence, health governance, and low- and middle-income countries [14]. Grey literature was retrieved from authoritative sources such as the World Health Organization (WHO), the Global Polio Eradication Initiative (GPEI), the Global Fund to Fight AIDS, Tuberculosis, and Malaria, and other major international health agencies [15]. Policy analyses and cost estimates were included to provide contextual and economic perspectives.

Inclusion and Exclusion Criteria

Diseases were included if they met two criteria: (1) the availability of proven, cost-effective interventions capable of achieving eradication or substantial burden reduction; (2) demonstrated feasibility of implementation in controlled or real-world settings.

Conditions for which no proven intervention exists, or for which eradication is biologically implausible, were excluded [16,17].

Search Results and Synthesis

Our database searches of PubMed, Web of Science, and Scopus, together with targeted grey literature searches from the WHO, the Global Fund, CDC, and the GPEI, yielded approximately 1,500-1,700 records. After screening titles and abstracts for relevance to eradicable or highly reducible diseases and AI/global health governance, we reviewed about 120 full-text sources. Of these, 46 peer-reviewed articles and 12 grey literature or policy reports were included in the final narrative synthesis, along with methodological references such as the Cochrane Handbook and the WHO evidence synthesis guides.

Data Extraction and Synthesis

For each included condition, data were extracted on global burden measured in disability-adjusted life years (DALYs) or mortality, estimated annual eradication or control costs, and documented barriers to implementation. Where possible, data were cross-referenced among multiple sources to enhance reliability. A thematic synthesis approach was applied to identify recurring structural barriers, including political, governance, health system, and sociocultural factors [16,18].

Conceptual Framework

The synthesis was guided by a framework conceptualizing health outcomes as the product of three interrelated components: technological capacity, political will, and system readiness. This framework informed the interpretation of the findings and the assessment of whether future technologies, such as AI, are likely to achieve equitable health impact without accompanying structural reforms [19-21].

Results

Classification of Conditions

Analysis of literature and global health data is done in two principal categories: eradicable diseases and highly reducible or preventable conditions.

Eradicable Diseases

These are conditions for which global elimination is technically feasible with existing tools. These include poliomyelitis, measles, rubella, malaria, tuberculosis, lymphatic filariasis, obstetric fistula, and severe acute malnutrition. All have well-established, cost-effective interventions such as vaccines, antimicrobial therapies, surgical procedures, or comprehensive public health programs. The global polio eradication effort has sustained transmission interruption in multiple regions, while measles elimination has occurred in countries with high vaccination coverage [22,23]. Malaria control programs combining vector management, rapid diagnostics, and artemisinin-based combination therapy have yielded substantial declines in incidence where consistently applied [24,25]. However, uneven implementation, funding gaps, and weak health systems have allowed these diseases to persist in specific geographic and socio-political contexts [26].

Highly Reducible or Preventable Conditions

These are conditions for which widespread implementation of proven interventions could reduce the global burden by at least 80%. This includes cervical cancer, hepatitis B and C, maternal mortality, neonatal deaths, diarrheal diseases, respiratory infections, mother-to-child HIV transmission, rheumatic heart disease, and cataract blindness. Proven prevention and treatment strategies such as vaccination, antenatal care, skilled birth attendance, surgical interventions, and safe water and sanitation are widely available but remain inaccessible to many due to inequities in resource distribution, workforce shortages, and sociocultural barriers [27-30].

Comparative Burden and Feasibility

Table 1 summarizes selected conditions, their annual global DALY burden, estimated cost of eradication or substantial control, and feasibility with current tools. These financial requirements are modest compared with global spending in other sectors. For instance, the combined annual eradication costs for several high-burden diseases are equivalent to only a few weeks of global military expenditure [27,31].

**Table 1:** Selected conditions, global burden (DALYs), estimated eradication or control costs, and feasibility with current tools. Data sources: World Health Organization (WHO) Global Health Observatory, Global Burden of Disease Study 2021, and published eradication cost estimates [30-35]. Global Burden of Disease Study 2021 (DALYs, Institute for Health Metrics and Evaluation); WHO Global Health Observatory (2019-2023 estimates); Global Polio Eradication Initiative (2019-2023) for polio costs; Coates et al. [37] (Lancet Global Health, 2021) for malaria and rheumatic heart disease; WHO Tuberculosis Fact Sheet (2025) for TB funding; Kastner et al. [35] (PLoS Negl Trop Dis, 2017) for lymphatic filariasis; UNFPA program reports (2022) for obstetric fistula; Carter Center/WHO (2019) for Guinea worm eradication; UNICEF “No Time to Waste” (2020) for severe acute malnutrition; Kocarnik et al. [30] (JAMA Oncology, 2022) for cervical cancer; Buckley & Strom [36] (National Academies, 2017) for hepatitis B & C; Jamison [27] (Disease Control Priorities in Developing Countries, 2006) for maternal and child survival interventions (used here as conservative cost benchmarks); Oza et al. [28] (Bull WHO, 2015) for neonatal causes; WHO/UNAIDS (2019-2022) for HIV mother-to-child transmission; and WHO blindness program reports (2018–2022) for cataract surgery costs. DALY: disability-adjusted life year; GPEI: Global Polio Eradication Initiative; LMICs: low- and middle-income countries; UNFPA: United Nations Population Fund; UNICEF: United Nations Children's Fund.

Category	Condition	DALYs (millions)	Estimated eradication/control cost (USD)	Feasibility with current tools
Eradicable	Polio	0.2	4.2 billion (total for completion, GPEI 2019–2023) [32].	High
	Measles & rubella	9.1	7.5 billion	High
	Malaria	45.9	6.5 billion annually (for control; eradication ~100 billion total 2015-2040) [33]	Moderate-high
	Tuberculosis	40.6	22 billion annually [34]	Moderate-high
	Lymphatic filariasis	1.2	0.9 billion (total for elimination) [35]	High
	Obstetric fistula	0.1-0.5	350-500 per surgery	High
	Guinea worm disease	<0.001	150 million (total for eradication)	Very high
	Severe acute malnutrition	30.2	10 billion annually	High
Highly reducible/preventable	Cervical cancer	8.2	10 billion	High
	Hepatitis B & C	15.8	11 billion annually [36]	High
	Maternal mortality	9.2	115 billion over 5 years	High
	Neonatal deaths	19.4	16 billion annually	High
	Childhood diarrhea & pneumonia	58.7	13 billion annually	High
	HIV mother-to-child transmission	1.3	3.2 billion annually	Very high
	Rheumatic heart disease	4.9	2.5 billion annually [37]	High
	Cataract blindness	15-18	25-100 per surgery (in LMICs)	Very high

Key Patterns

Across both categories, the evidence shows that technological sufficiency does not guarantee implementation success. The limiting factors are political will, governance quality, and health system readiness, rather than scientific or technical feasibility. Regions with strong health infrastructure and sustained political commitment have achieved or approached elimination, while areas with governance instability, chronic underfunding, or inequitable health systems have seen little progress despite having access to the same tools [38-41].

Discussion

This review highlights a paradox in global health: many diseases of poverty persist despite proven, cost-effective interventions capable of eradication or major burden reduction. The findings make clear that the primary barriers to elimination are not technological, but structural, rooted in political will, governance, and health system capacity.

Lessons From Historical Precedents

Historical eradication campaigns such as those for smallpox and rinderpest succeeded not merely because of technological innovation, but because of sustained political commitment, coordinated governance, and strategic resource mobilization [42,43]. In contrast, diseases such as poliomyelitis and measles remain endemic in certain regions despite decades of vaccine availability. This illustrates that even the most effective biomedical tools can fail when delivery systems are weak, public trust is low, or political priorities shift away from public health.

The repeated underutilization of existing tools offers a sobering forecast for the deployment of future innovations, including AI. While AI holds promise for advancements in diagnostics, surveillance, and resource allocation, its benefits will remain unevenly distributed without structural reforms that address the inequities and inefficiencies embedded in current health systems [44,45].

The Role of Political Will and Governance

Political will is the decisive factor in translating technological capacity into population-level health outcomes. Countries that have aligned political priorities with public health goals through stable funding, transparent governance, and community engagement have made substantial progress toward eradication [46,47]. Conversely, in settings where health policies are fragmented, funding is unreliable, and accountability mechanisms are weak, the same technologies have produced minimal gains [48].

Governance is equally critical. Successful eradication requires coordinated strategies that connect ministries of health, finance, and education, and that engage civil society in both design and delivery. Without such integration, technological tools are often deployed in fragmented, duplicative, or unsustainable ways [48,49].

Implications for the AI Era

The global health community risks repeating historical patterns if AI is introduced into environments unprepared to use it effectively. Assuming innovation automatically improves outcomes overlooks the political, economic, and social determinants shaping health service delivery. In fact, AI could widen existing inequities if deployed primarily in high-resource settings while low-resource regions continue to struggle with basic healthcare provision [44,50,51].

Concrete examples illustrate both the promise and the risks of repeating historical patterns when deploying AI technologies in global health. WHO-recommended computer-aided detection tools such as CAD4TB, qXR, and Lunit INSIGHT CXR now meet the target criteria for triage accuracy in low-resource tuberculosis screening settings [52]. In malaria diagnosis, deep learning models, including YOLOv5 and other object-detection approaches, have achieved accuracy levels comparable to expert microscopists (~98%) [53]. In maternal health, machine learning models for preeclampsia and obstetric risk piloted in LMICs such as Ethiopia have demonstrated strong predictive performance [54]. At the planetary scale, outbreak surveillance platforms like BlueDot and HealthMap detected the spread of COVID-19 ahead of official reports, underscoring AI’s potential role in early-warning systems [55]. However, the success of these AI tools is contingent not only on algorithms but critically on underlying health system readiness, governance, and equitable implementation, falling short without structural reform.

Economic Considerations

The cost of eradicating many diseases of poverty is modest compared to global expenditures in other sectors. For example, the annual investment required to eliminate several high-burden conditions is equivalent to only a few weeks of global military spending [31]. This disparity underscores that the obstacle is not affordability, but rather the prioritization of health within national and international policy frameworks.

Limitations

This review is limited by its reliance on available global burden and cost estimates, which may vary in accuracy across sources. Additionally, while thematic synthesis identifies structural barriers, the relative weight of each barrier likely differs by disease context and region. Future research should incorporate region-specific analyses and policy case studies to refine these findings and support targeted recommendations.

## Conclusions

The eradication of many diseases of poverty is both technically and financially feasible with tools currently available. Yet history demonstrates that technological capability alone is insufficient to achieve population-wide health gains without sustained political commitment, accountable governance, and equitable health system capacity. The persistent underutilization of proven interventions is the most reliable predictor of how future innovations, including AI, will be deployed.

Closing the gap between what is possible and what is achieved requires reframing disease eradication as a political and moral imperative rather than a purely technological challenge. Without structural reforms to strengthen health systems, ensure equitable resource distribution, and embed accountability at every level of governance, AI and other emerging technologies will not succeed where existing solutions have failed. The true test for the global health community is the will to implement the tools already within reach. If we cannot use what we have now, we will not use what we get in the future.
